# The Bookworld of Medicine and Science

**Published:** 1905-06-03

**Authors:** 


### THE BOOKWORLD OF MEDICINE AND SCIENCE.

CASE-TAKING FORMS AND CHARTS FOR USE IN SANATORIA FOR CONSUMPTIVES. (London: The Scientific Press, 28 and 29 Southampton Street, Strand, W.C.)

THE case-forms commence with the usual headings for name, address, etc., followed by spaces for the "History of the Present Illness and Previous History." Various other useful data are to be put down seriatim, while there is ample room for the physical signs and chest-outlines for graphic representation. The progress of the case is provided for under headings of Date, Notes, Treatment, and Diet. The temperature chart of corresponding size, gives on the one margin the Fahrenheit reading, on the other the Centigrade. We are glad to note that daily space is provided for exercise, work, rest, baths, etc. Finally, there is a good chart for post-mortem findings and records. We consider that it is a matter of no small moment that a uniform system of note-taking should be utilised in sanatoria generally, so that the same series of data should be available for the purpose of collective investigation and for comparing results, and it would be of very great assistance if a number of the more important sanatoria would agree on a common system that should be available for all such institutions. The charts examined are excellent, but perhaps might be still further improved. For instance, outlines of the laryngoscopic image should be included for recording exactly the laryngeal condition. A chest-outline stamp* should likewise be obtainable to correspond with the initial record, so that weekly or monthly variations should be duly recorded. Moreover, there should be a uniform system of signs or markings to indicate physical signs, *e.g.* dulness, fine crepitations, rales, harsh-breathing, etc., and these signs should be stated on any system of charts which are intended for general usage. The case-forms and the charts are in use at the Mount Vernon and Northwood Consumption Hospitals, and are the result of Dr. Kelynack's practical experience of the needs of such institutions.

* The Publishers announce that they supply the indiarubber stamp recommended by the reviewer.

THE ARCHIVES OF MIDDLESEX HOSPITAL, VOL. IV. (London: Macmillan and Co. 1905. Pp. 159. Illustrations 19, plates 12.)

THIS volume, in addition to the reports of the various registrars, contains the following articles:—"Tubal Gestation: a pathological study by Comyns Berkeley and Victor Bonney"; "The Surgical Anatomy of the Operation of Suprapubic Prostatectomy, by Thomson Walker"; "Notes on Three Cases of the Rarer Varieties of Anæmia, by A. K. Williamson"; "The Microscopic Examination of the Spinal Cords from Two Cases of Landry's Paralysis, by John J. Douglas"; "Relationship of Broad Ligament Cysts to Hydrosalphinx, by Sampson Handley; "Description of a New Form of Warm Stage," by W. T. Hillier.

